# Effects of Multidimensional Carbon-Based Nanomaterials on the Low-Carbon and High-Performance Cementitious Composites: A Critical Review

**DOI:** 10.3390/ma17102196

**Published:** 2024-05-08

**Authors:** Xiumei Gao, Wujun Fang, Weiwen Li, Peng Wang, Kashan Khan, Yihong Tang, Teng Wang

**Affiliations:** 1 College of Civil & Transportation Engineering, Shenzhen University, Shenzhen 518060, China; xm08081994@163.com (X.G.); fangwujun@cribc.com (W.F.); 2 MCC Group, Central Research Institute of Building and Construction (Shenzhen) Co., Ltd., Shenzhen 518055, China; 3 Department of Civil Engineering, Tianjin University, Tianjin 300072, China; kashan@tju.edu.cn; 4 Department of Civil and Environmental Engineering, The Hong Kong University of Science and Technology, Hong Kong 999077, China; ytangck@connect.ust.hk; 5 Department of Civil Engineering, The University of Hong Kong, Hong Kong 999077, China; wangteng@connect.hku.hk

**Keywords:** cementitious composites, carbon-based nanomaterials, low carbon, high performance, multidimensional effects

## Abstract

Cementitious composites are ubiquitous in construction, and more and more research is focused on improving mechanical properties and environmental effects. However, the jury is still out on which material can achieve low-carbon and high-performance cementitious composites. This article compares the mechanical and environmental performance of zero-dimensional fullerenes, one-dimensional carbon nanotubes (CNTs), two-dimensional graphene oxide (GO), and three-dimensional nano-graphite platelets (NGPs) on cementitious composites. The literature review shows that two-dimensional (2D) GO has the best mechanical and environmental performance, followed by 3D NGPs, 1D CNTs, and 0D fullerenes. Specifically, GO stands out for its lower energy consumption (120–140 MJ/kg) and CO_2_ emissions (0.17 kg/kg). When the optimal dosage (0.01–0.05 wt%) of GO is selected, due to its high specific surface area and strong adhesion to the matrix, the compressive strength of the cementitious composites is improved by nearly 50%. This study will help engineers and researchers better utilize carbon-based nanomaterials and provide guidance and direction for future research in related fields.

## 1. Introduction

Cementitious composites are composed of amorphous phases, nanocrystals to micrometer-sized crystals, and bound water [1]. These materials have excellent compressive strength and durability but often exhibit brittleness and lack sufficient tensile strength and strain capacity [2,3]. In order to overcome these limitations, researchers have been exploring the effect of reinforcing fillers to improve the toughness and strength of cementitious composites. In recent years, research has shown that as the size of fillers decreases, from macro- to micro- and even nano-levels, people are surprised to find that the addition of small fillers not only improves the mechanical properties and durability of cementitious materials but also endows them with multifunctionality [4,5]. The research indicates that by adding nano-silica particles, the compressive strength of concrete can be increased by up to 70% [6]. At the same time, the addition of only 5% nano-alumina can increase the elastic modulus of cementitious composite materials by up to 143%, indicating a significant improvement in material properties by nano-fillers [7]. In addition, the study also pointed out the potential applications of nano titanium dioxide. The addition of this nanomaterial gives concrete many additional functions, including self-cleaning, reducing air pollution, and sterilization [8].

**Figure 1:**
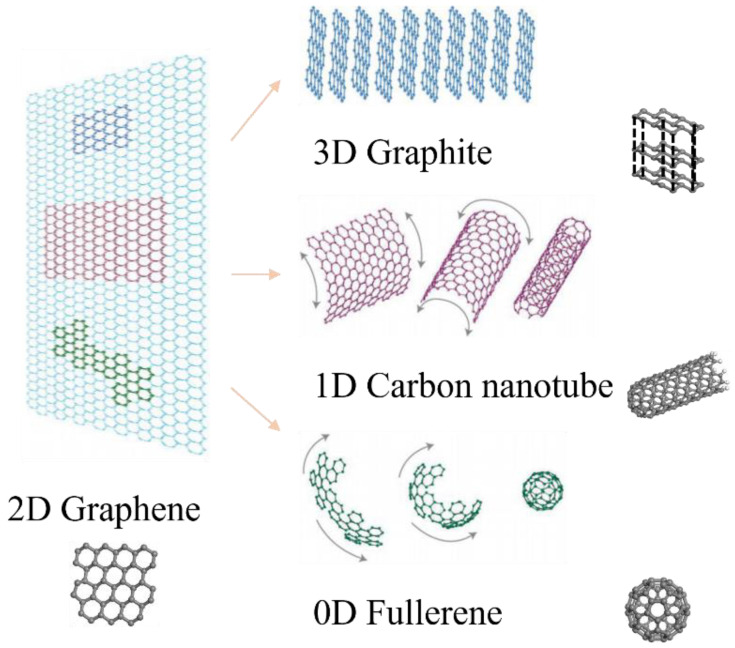
Graphene: wrapped up into 0D, rolled up into 1D, stacked into 3D [12].

Since 2004, with the rapid development of nanotechnology and science, graphene has received great attention at the nanoscale and has shown great potential as an additive material [9]. The preparation process of graphene can be summarized as follows: gaseous carbon sources such as methane or ethylene are used to catalyze the deposition and polymerization of carbon atoms on the surface of metal (nickel, copper, or platinum). Equations (1) and (2), respectively, indicate the chemical reaction in which methane or ethylene is used as a gaseous carbon source. Graphene is a single-layer graphite structure formed by carbon atoms arranged into a 2D lattice. It is one of the basic components of graphite materials. In addition to single-layer graphene, graphene can also form various forms, such as fullerene, carbon nanotube, and graphite [10,11] (as shown in Figure 1). The various forms of graphene and its derivatives provide rich material resources and technical means for scientific research and engineering applications in various fields.

(1) $$CH_{4}\to C(graphene)+2H_{2}$$

(2) $$C_{2}H_{4}\to C(graphene)+2H_{2}$$

Nowadays, sustainable development has become one of the focal points of global attention. The selection of building materials directly affects the environmental friendliness, resource utilization efficiency, and long-term sustainability of buildings. The policies and requirements for reducing energy consumption and reducing waste generation are all aimed at achieving the sustainable development goals of the construction industry. According to previous research [13], the cement production industry not only accounts for a considerable proportion of energy consumption, accounting for approximately 7% of total energy consumption, but also plays an important role in carbon dioxide emissions, accounting for nearly 7% of total carbon dioxide emissions.

The current research urgently needs to review the existing achievements, especially emphasizing the improvement of mechanical properties and environmental effects of carbon-based nanomaterials in cementitious composites and further elucidating their mechanisms of action. The purpose of this survey is to comprehensively summarize previous research results and explore, in-depth, the performance improvement of carbon-based nanomaterials in cementitious composites. Through the summary and analysis of these studies, we can better understand the mechanism of carbon-based nanomaterials in cement-based composites. The suggested research will help engineers and researchers better utilize carbon-based nanomaterials as reinforcing materials and provide guidance and direction for future research in related fields.

## 2. Zero-Dimension Nanocarbon Material

### 2.1. Fullerenes

Fullerenes are molecules composed of carbon atoms with spherical, tubular, or other geometric shapes [14,15,16]. The discovery of fullerene opened a new chapter in the field of carbon-based nanomaterials in 1985 [17].

**Figure 2:**
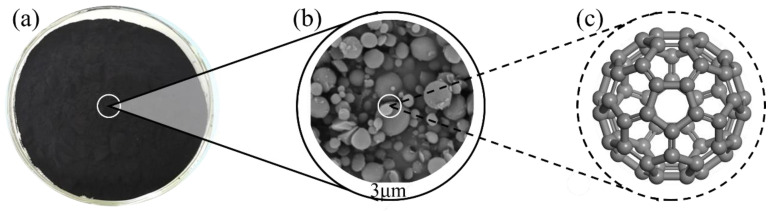
Properties of C_60_ fullerenes. (**a**) Macroscopic appearance; (**b**) SEM images; (**c**) molecular structure.

C_60_ fullerene is the most typical member of the fullerene family and was also the earliest discovered [18]. As shown in Figure 2, C_60_ fullerene is a spherical carbon molecule that exhibits a geometric shape like a football. C_60_ fullerene typically exhibits a black color [19]. Each C_60_ fullerene molecule is composed of 12 regular pentagons and 20 regular hexagons, and this structure is called a “pentagonal hexagonal combination” [20]. Each carbon atom forms covalent bonds with three adjacent carbon atoms [21], forming a spherical carbon molecular structure. The arrangement of these carbon atoms gives C_60_ fullerene a high degree of symmetry and stability [22].

### 2.2. Manufacturing Process

**Figure 3:**
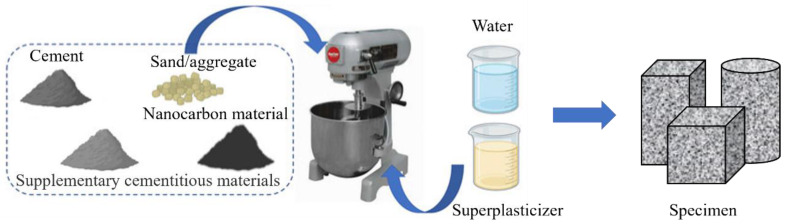
The mixing process of cementitious composites incorporated nanocarbon material.

The components of cementitious composites include Portland cement, quartz sand, water, and additives. Carbon-based nanomaterials are usually mixed into the matrix as additives [23,24,25]. The preparation process of carbon nanomaterial-based cementitious composites is shown in Figure 3. Firstly, we mixed the weighed dry material at low speed in the mixer for 4 min. Then, we added water and liquid additives to the dry mixture, stirred at low speed for 1 min, and then stirred at high speed for 2 min. Subsequently, we added fibers and continued at low speed for 1 min. After the fibers were completely wrapped in the cement slurry, we mixed them at high speed for 4 min until the fibers were evenly dispersed in the cement slurry. 

This article not only focuses on the preparation process of nanocarbon material-based cementitious composites but also on the energy consumption, CO_2_ emissions, cost, time, and water demand during this process. In the preparation process of nanocarbon material, energy consumption and CO_2_ emissions are closely related, which has a significant impact on the environment. The cost is mainly affected by energy consumption, raw material costs, labor costs, and time. Water demand is affected by using water as a reaction medium or cleaning agent during the preparation process, which is of great significance for resource utilization and environmental protection. When the matrix material is consistent, we should focus on carbon-based nanomaterials. According to existing literature reports and experimental data [26,27,28], producing 1 kg of C_60_ fullerene may require energy of 2478 MJ, emit 400 kg of carbon dioxide, and cost range from USD 150 to USD 1080. Because the preparation process of C_60_ fullerene is relatively complex, involving multiple steps such as carbon source pyrolysis, carbon atom polymerization, and subsequent purification and treatment, it may take several weeks to produce 1 kg of C_60_ fullerene [29]. However, the water requirement during the preparation process is relatively low, usually ranging from tens to hundreds of liters.

### 2.3. Mechanical Properties

At present, research on cementitious composite materials constructed directly from 0D fullerene is relatively scarce [30]. This may be due to some challenges of 0D fullerene in cementitious composites, especially the lack of ability to prevent microcracks, which may lead to the formation of weak regions [31]. In contrast, similar 0D carbon black (CB) particles are relatively more cost-effective, which makes people more inclined to choose CB as an additive for cementitious composites [32,33,34]. Adding CB appropriately can not only improve mechanical strength to a certain extent but also have the potential to be used for strain sensing [34,35,36]. Although 0D fullerene has great potential in structural health monitoring, its high price limits its widespread application in cementitious composite materials [37]. Therefore, future research may need to explore more cost-effective preparation methods and customized solutions for price-sensitive applications in order to promote the development and application of this field.

## 3. One-Dimension Nanocarbon Material

### 3.1. Carbon Nanotubes

**Figure 4:**
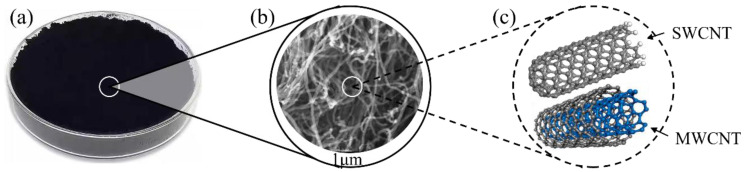
Properties of carbon nanotubes. (**a**) Macroscopic appearance; (**b**) SEM images;(**c**) molecular structure.

CNT-based cementitious composites have been widely studied, mainly due to the specific properties and structure of carbon nanotubes [38]. CNTs are 1D nanoscale structures composed of carbon atoms, divided into single-walled carbon nanotubes (SWCNTs) and multi-walled carbon nanotubes (MWCNTs) [39,40]. As demonstrated in Figure 4, CNTs exhibit a slender tubular structure at the macroscale, with lengths ranging from several micrometers to several centimeters and diameters typically within the nanoscale range [41]. From the perspective of molecular structure, SWCNTs are composed of a single layer of carbon atoms arranged in a continuous hexagonal structure resembling a coiled graphene sheet. MWCNTs are composed of multiple concentric carbon layers, each connected by a meta-like bond [42]. The carbon atoms of carbon nanotubes exhibit sp^2^. Hybridization forms a π bond network with strong conjugation [43,44,45]. This network alters the surface properties of CNTs, which results in improved adhesion characteristics and dispersion stability. Hence, carbon nanotubes have good conductivity and mechanical properties.

### 3.2. Manufacturing Process

Different production processes can result in varying energy consumption per kilogram of carbon nanotubes [46]. The production process of CNTs includes steps such as pyrolysis of carbon raw materials [47], gas-phase nucleation, and growth [48], all of which require a considerable amount of energy consumption. It is estimated that producing 1 kg of carbon nanotubes may require 1800 MJ of energy and emits 125 kg of carbon dioxide [26,49]. The production cost of carbon nanotubes is also relatively high, as the production process involves complex chemical reactions, high-temperature conditions, and precision instruments [50]. It is estimated that the cost of producing 1 kg of carbon nanotubes may be USD 369 [51]. The production time of 1 kg carbon nanotubes is also relatively long, requiring precise equipment and control conditions. Producing 1 kg of carbon nanotubes may take one week [29]. The water used in the production of carbon nanotubes is used for equipment cleaning and the use of some solvents. Therefore, the water requirement for producing 1 kg of carbon nanotubes may also range from tens to hundreds of liters.

### 3.3. Mechanical Properties

Numerous researchers have studied how to improve the performance of cementitious composites by changing the percentage of carbon-based nanomaterials in cement weight. A large number of experimental results [52,53,54,55,56,57,58,59,60,61] indicate that the compressive strength of cementitious composites with CNTs shows a trend of first increasing and then decreasing with the increase in CNT content. The cementitious composites doped with CNTs can significantly improve the conductivity, with a typical penetration threshold between 0.3 and 0.6 wt% [62,63,64]. However, in terms of the compressive strength of cementitious composites, the optimal amount of CNTs is generally 0.01–0.15 wt%, and the maximum improvement rate is generally 30%. The determination of this optimal value is related to a study that found that with the addition of 0.1 wt% CNTs, the enthalpy of cement paste is 20% lower than that of pure cement paste [59]. This indicates that CNT particles form a package around cement particles, causing some cement particles to separate during the hydration process [65]. However, at higher doses of CNTs, this effect may inhibit cement hydration, thereby reducing bonding strength [59]. Overall, the amount of CNTs added should be within a certain range, and excessive or insufficient amounts may affect the performance of cementitious composites.

**Figure 5:**
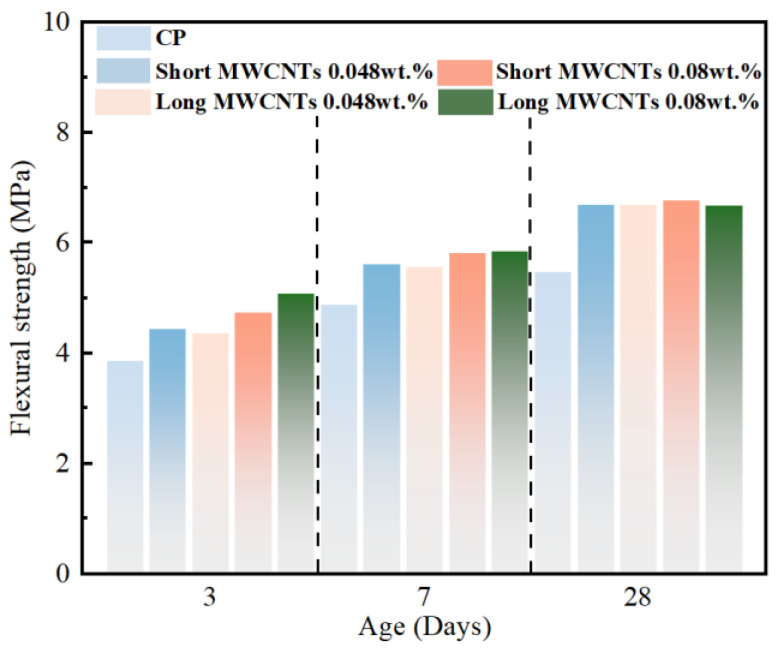
Effect of different types (short and long) of MWCNTs and concentration on the flexural strength [69].

In addition, some researchers have also focused on the flexural performance of cementitious composites containing CNTs. Zou et al. [66] have shown that the elastic modulus and flexural strength of cementitious composites increase at concentrations of 0.075 wt% and 0.038 wt%. Another study [67] explored the impact of incorporating long CNTs of 0.1, 0.5, and 1.0 wt% with silica fume on the properties of cementitious composites. On the 28th day, their findings indicated that the most significant enhancements in both flexural strength and stress-intensity factor were achieved through the addition of 0.5 wt% CNTs to the cement mix. As shown in Figure 5, Maria S. Konsta Gdoutos et al. [68] enhanced the strength of cementitious composites by 62.5% and 56.25%, respectively, by adding 0.08 wt% short MWCNTs and 0.048 wt% long MWCNTs. 

## 4. Two-Dimension Nanocarbon Material

### 4.1. Graphene

**Figure 6:**
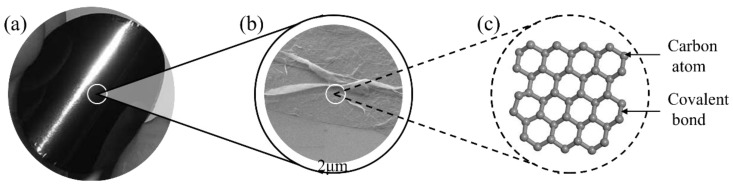
Properties of graphene. (**a**) Macroscopic appearance; (**b**) SEM images; (**c**) molecular structure.

In 2004, Novoselov and Geim et al. used a method called “mechanical exfoliation” to successfully prepare graphene monolayers by peeling graphite sheets with tape [9]. Graphene is a single-layer 2D structure material composed of carbon atoms, and its unique properties and structure make it a research hotspot in the field of nanotechnology [69]. Figure 6 depicts the macroscopic appearance, micromorphology, and molecular structure of graphene. Graphene exhibits a transparent and colorless appearance at the macroscopic scale, with a thickness of only one atom. The theoretical surface area of a single graphene sheet can reach 2600 m^2^/g [70,71,72,73]. The microstructure of graphene is characterized by a hexagonal lattice structure formed by covalent bonds of carbon atoms [74]. The arrangement of carbon atoms is very ordered and flat, which leads to excellent electrical, thermal, and mechanical properties in a single-layer state. The preparation methods of graphene include mechanical exfoliation [75], chemical vapor deposition [76], and liquid-phase exfoliation [77].

### 4.2. Manufacturing Process

The preparation methods of graphene include chemical vapor deposition (CVD), mechanical exfoliation, chemical exfoliation, reduced GO, and liquid-phase exfoliation [78]. Among them, the CVD method is a common and widely used method, which forms graphene by cracking carbon source gas at high temperatures and depositing it on a metal substrate [79]. The CVD method typically requires a high-temperature reaction environment, which consumes a significant amount of energy [80]. Specifically, the energy consumption for preparing graphene by CVD method is 120–140 MJ/kg of graphene [81,82,83]. The CVD method for preparing graphene typically uses hydrocarbon gases as a carbon source; the preparation of graphene per kilogram may result in emissions of 0.17 kg of carbon dioxide [84,85]. In the CVD method, the main sources of cost are gas, energy, and equipment maintenance. Specifically, the preparation cost of graphene per gram may be USD 35 [86]. The preparation process of graphene generally does not require a large amount of water and is mainly used for cleaning and solvent treatment. For the most part, the entire growth process from heating to cooling may take 5–48 h [87,88,89].

### 4.3. Mechanical Properties

Graphene is the fundamental structural unit of any size of graphite material. GO, the most researched graphene-based nanosheets in cement composites also consists of monolayer sheets with a hexagonal carbon network. The laboratory data reported in the literature, as shown in Table 1, indicate that adding a small amount of GO to cementitious composite materials will enhance their flexural, compressive, and tensile strength. Lv et al. [90,91] conducted a study focusing on the effect of GO in a cement matrix. Their research indicates that as the proportion of GO increases to 0.03%, the performance of the cement matrix is enhanced. However, it is worth noting that further increasing the proportion of GO may lead to a decrease in the strength effect. Specifically, at a ratio of 0.03 wt% GO, the tensile, bending, and compressive properties of the cement matrix were improved by 78.6%, 60.7%, and 38.9%, respectively. In addition, Duan et al. [3,92] confirmed the reinforcing effect of GO in a cement matrix. In an ordinary Portland cement (OPC) matrix, using only 0.05 wt% graphene oxide nanosheets can increase the bending and compressive strength by 41–59% and 15–33%, respectively.

Furthermore, Jiang et al. [93] investigated the effect of combined modification of polyvinyl alcohol (PVA) fibers and GO on the mechanical properties of cement mortar in their experiments. The addition of PVA fibers significantly improves the toughness and fracture resistance of mortar, thereby significantly enhancing its mechanical strength [94]. Meanwhile, the cementitious matrix system containing GO improved the pore structure of the mortar, exhibiting improved mechanical and durability characteristics [95]. Jiang et al. conducted experiments to explore the effect of combined modification of PVA fiber and GO on the mechanical properties of cementitious composites. The experimental results showed that the compressive and flexural strength of mortar increased by 30.2% and 39.3%, respectively, after adding PVA fibers and graphene oxide [93].

**Table 1:** Effect of GO on mechanical performance of cementitious composites.

Serial Number	Compressive Strength		Flexural Strength		Tensile Strength		Water/ Binder	Refs.
	GO (wt%)	Increase (%)	GO (wt%)	Increase (%)	GO (wt%)	Increase (%)		
1	0.01	5.16	0.03	21.86	-	-	0.35	[95]
2	0.01	13.4	0.01	51.7	0.01	47	0.367	[90]
3	0.01	29	-	-	0.01	26	0.5	[96]
4	0.02	23.2	-	-	0.04	38.5	0.43	[97]
5	0.02	20	0.02	32	-	-	0.5	[98]
6	0.02	27.64	-	-	-	-	0.5	[99]
7	0.02	25	0.02	15	0.02	15	0.4	[100]
8	0.02	25.9	0.02	14.8	0.02	18	0.4	[101]
9	0.022	34.1	0.022	34	-	-	0.4	[102]
10	0.022	27	0.022	26	-	-	0.42	[103]
11	0.022	25.6	-	-	-	-	0.29	[104]
12	0.022	25.8	-	-	-	-	0.36	[104]
13	0.022	24.6	-	-	-	-	0.45	[105]
14	0.025	14.9	0.025	23.6	0.025	15.2	0.5	[106]
15	-	-	0.03	13.7	-	-	0.43	[107]
16	0.03	38.9	0.03	60.7	0.03	78.6	0.367	[90]
17	0.03	20.3	0.03	32	-	-	0.5	[108]
18	-	-	0.03	77.7	-	-	0.36	[109]
19	0.03	12.4	0.03	12.08	-	-	0.4	[110]
20	0.03	45.1	-	-	0.03	60.7	0.37	[111]
21	0.03	30	0.03	18.7			0.45	[91]
22	0.03	31	0.03	18			0.45	[112]
23	0.03	28	-	-	-	-	0.36	[113]
24	0.04	13.4	-	-	0.04	9.9	0.4	[114]
25	0.04	44	-	-	-	-	0.38	[115]
26	0.04	40.41	-	-	-	-	0.4	[116]
27	0.04	29.3	0.04	15	0.04	15	0.4	[117]
28	0.04	42.2	0.04	30.5	0.04	36.6	0.367	[90]
29	0.04	46.34	-	-	-	-	0.5	[99]
30	0.04	47.61	-	-	-	-	0.36	[109]
31	0.044	29.5	-	-	-	-	0.5	[104]
32	0.05	24.4	0.05	70.5	-	-	0.37	[118]
33	0.05	32	-	-	-	-	0.5	[119]
34	0.05	43.2	0.05	106.4	-	-	0.37	[111]
35	0.05	47.9	0.05	30.2	0.05	35.8	0.367	[90]
36	0.05	24.4	0.05	70.5	-	-	0.37	[120]
37	0.05	32	-	-	-	-	0.5	[12]
38	0.05	11.05	0.05	16.1	-	-	0.4	[110]
39	0.06	29.5	0.06	30.7	-	-	0.3	[121]
40	0.1	13	0.01	23.4	-	-	0.48	[122]
41	0.1	77.7	0.1	77.7	0.1	37.5	0.485	[123]
42	0.125	40	-	-	-	-	0.45	[124]
43	0.125	35.1	-	-	0.125	96	0.45	[125]
44	0.125	110	-	-	-	-	0.45	[126]
45	0.2	16.4	0.2	41.3	-	-	0.66	[127]
46	0.5	126.6	-	-	-	-	0.3	[128]
47	0.5	126.6	-	-	-	-	0.3	[129]
48	1.0	77.8	-	-	-	-	0.45	[126]
49	1.0	63	-	-	-	-	0.45	[130]
50	1.0	77.3	-	-	1.0	15	-	[131]
51	1.0	86	-	-	1.0	15	0.45	[96]
52	-	-	-	-	1.5	48	0.4	[2]
53	-	-	1.5	51.2	-	-	0.3	[132]

## 5. Three-Dimension Nanocarbon Material

### 5.1. Graphite

**Figure 7:**
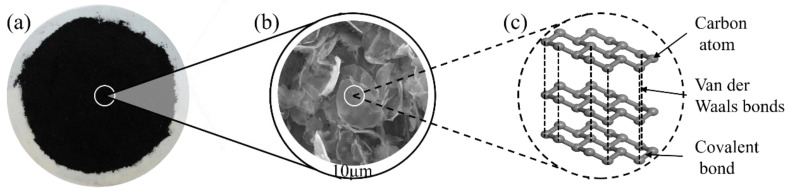
Properties of graphite. (**a**) Macroscopic appearance; (**b**) SEM images; (**c**) molecular structure.

Graphite is a mineral composed of carbon atoms and is one of the allotropes of non-metallic elements [94,133]. For graphite, its elastic modulus is usually around 1 TPa, which indicates that graphite has very high stiffness and bending resistance [110,134]. Figure 7 depicts the macroscopic appearance, micromorphology, and molecular structure of graphite. Graphite usually presents a black to silver-gray appearance, with luster and metallic luster [135]. It can exist in the form of flakes or powders [136], and flake graphite exhibits a typical layered structure that can be easily peeled off into thin sheets. The microstructure of graphite is composed of multiple layers of graphene sheets [137]. Each layer of graphene sheet is composed of a hexagonal lattice of carbon atoms arranged in a plane, forming a 2D structure [138]. The molecular structure of graphite is composed of carbon atoms, each of which forms three covalent bonds and is connected to the surrounding three carbon atoms, forming a hexagonal circular structure. This hexagonal structure combines with Van der Waals forces in the plane, giving graphite a layered structure [139].

### 5.2. Manufacturing Process

NGP_S_ are one of the commonly used nanoscale graphite materials in cementitious composites. The thickness of NGP_S_ is usually between a few to tens of nanometers, while the length and width can reach several micrometers [140]. Pyrolysis is one of the methods for preparing NGP_S_ from natural graphite. This typically involves exposing natural graphite to high-temperature conditions, typically between 1000 and 3000 °C, and operating in an inert atmosphere such as nitrogen or argon [141]. This high-temperature environment can cause the graphite structure to undergo a pyrolysis reaction, decomposing into smaller graphite flakes [142]. Therefore, in the process of preparing 1 kg of NGP_S_, the energy consumed is between 264 and 304 MJ, and 16 kg of CO_2_ is emitted [143,144]. In terms of cost, the estimated cost of producing one ton of nano-graphite sheets is between USD 1500 and USD 2000 [145]. The water demand is relatively low, about 100 to 200 L per ton of production. The entire production cycle may last several days, depending on the different production equipment and processes.

### 5.3. Mechanical Properties

**Figure 8:**
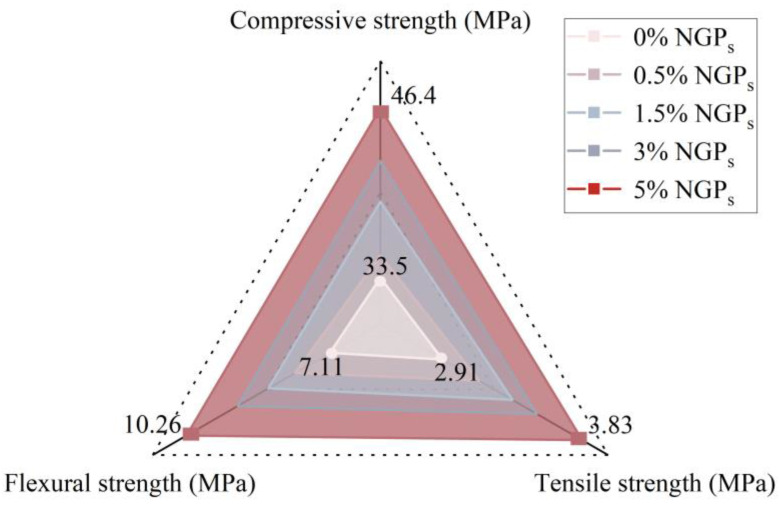
Effect of different contents of NGPs on mechanical strength of concrete [151].

NGP_S_ have shown significant potential. This material plays a crucial role in improving the “smart” performance of materials due to its unique structure and properties [146,147]. Sharma et al. [148] showed that the addition of NGP_S_ (i.e., 0.01%, 0.1%, and 0.2%) significantly improved the density and mechanical properties of concrete. The research results show that the density of concrete has increased by up to 16%, while the mechanical properties have increased by an astonishing 30%. It is worth noting that, in contrast to the increase in material strength and density, the permeability of concrete significantly decreases. Liu et al. [149] studied the effect of NGPs on cement mortar and reported a 36% increase in compressive strength. Yang et al. [150] conducted a study on the effect of NGP_S_ on the properties of cement mortar. By gradually increasing the amount of NGPs from 0.2% to 0.6%, researchers have found that when 0.2 wt% of NGPs are added to cement mortar, the compressive strength and flexural strength of cement mortar increase by about 10% and 8%, respectively. It is worth noting that research has also shown that cement mortar with the addition of NGPs has better acid resistance and durability. As shown in Figure 8, Farhan et al. [151] prepared five mixes with the intrusion of NGPs (0%, 0.5%, 1.5%, 3%, and 5% by weight of cement) to study workability and mechanical properties. The compressive strength, tensile strength, and flexural strength of the sample containing 5% NGPs increased by 38.5%, 31.6%, and 44.34%, respectively. 

## 6. Overall Assessment

### 6.1. Assessment of Property

**Figure 9:**
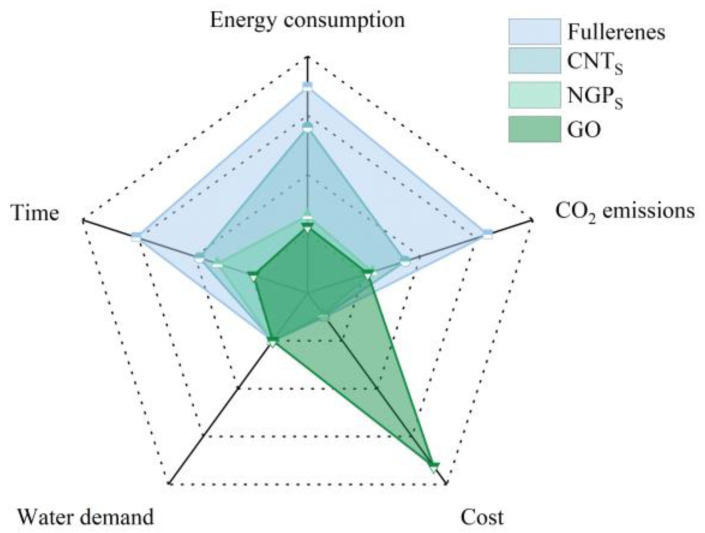
Comprehensive performance evaluation of carbon-based nanomaterials.

The selection of building materials has a significant impact on the environment. For cementitious composites, the improvement of strength is closely related to the hydration process of cement. However, cement production is a highly energy-consuming and high-emission activity process. Therefore, for carbon-based nanomaterials with small dosages but significant performance improvement, it is necessary to pay attention to their production process and evaluate them. Based on previous research work from the literature [26,27,28,29,46,47,48,49,50,78,79,80,81,82,83,84,85,86,87,88,89,140,141,142,143,144,145], a comprehensive evaluation of the environmental impact of carbon-based nanomaterials (fullerenes, CNT_S_, GO, and NGP_S_) in terms of energy consumption, CO_2_ emissions, cost, water demand, and time, is shown in Figure 9. From the four aspects of energy consumption, CO_2_ emissions, water demand, and time, GO is the worthiest of widespread promotion and use. However, the high cost limits its production. This arises from the high energy consumption, costly instrumentation, and intricate technological demands of procedures like CVD [152]. Currently, scholars [152,153] are advancing GO’s evolution through enhanced chemical treatment methodologies, the substitution of high-purity graphite with cost-effective alternatives, and the innovation of novel apparatus.

**Figure 10:**
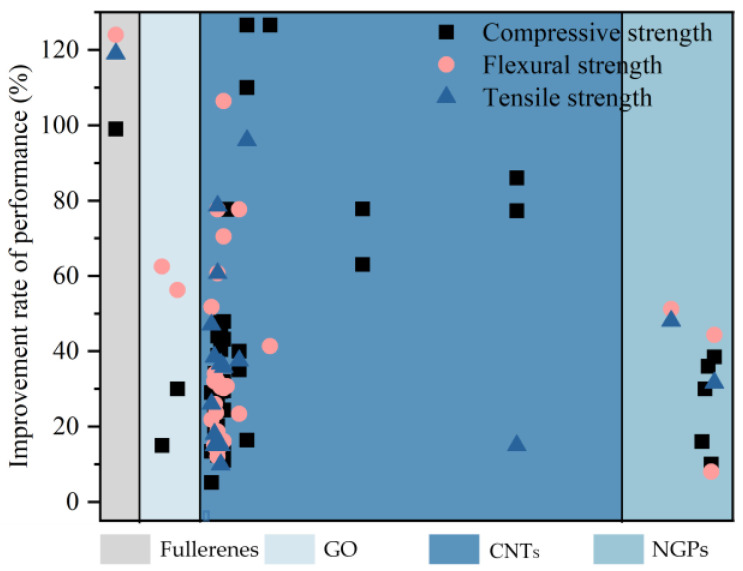
Summary of the mechanical properties of cementitious composites by different carbon-based nanomaterials.

Figure 10 depicts the impact of various carbon-based nanomaterials on the mechanical characteristics of cementitious composites. It is evident that the incorporation of carbon-based nanomaterials yields positive outcomes for cementitious composites. Although the mechanical properties of fullerenes (0D carbon-based nanomaterials) and CNTs (1D carbon nanomaterials) are significantly enhanced, they come at the expense of massive energy consumption, carbon emissions, and time passing. The improvement effect of NGPs on cementitious composites is still slightly inferior to that of GO. Consequently, current research primarily focuses on the influence of GO on cementitious composites. Additionally, it has been deduced that the optimal dosage for enhancing the compressive strength of cementitious composites ranges between 0.01% and 0.05%, with an approximate maximum enhancement rate of nearly 50%.

### 6.2. Analyses of Mechanism

**Figure 11:**
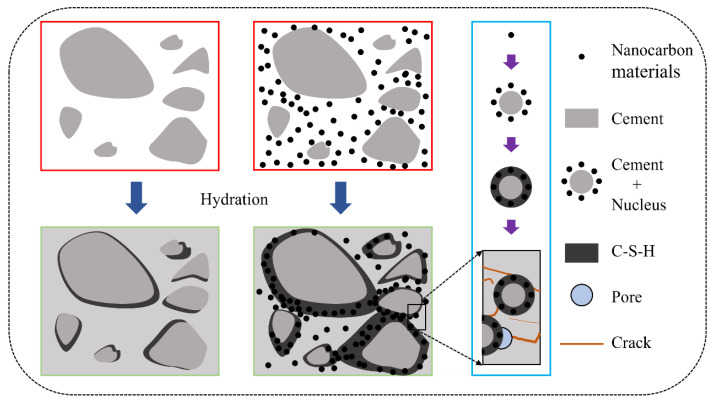
Schematic diagram of effect of carbon-based nanomaterials on the microstructures and cement hydration of the cementitious composites.

The mechanism by which multidimensional nanomaterials enhance the mechanical properties of cementitious composites can be summarized as nucleation effects and pore filling. The hydration process of cement can be divided into three stages: crystal growth, boundary reaction, and diffusion reaction [154]. Carbon-based nanomaterials can provide more nucleation centers during the crystal growth stage. On the one hand, nanomaterials can significantly accelerate the hydration reaction, thereby shortening the hardening time of cement and improving the early strength of cementitious composites. On the other hand, nanoscale hydration products can fill pores, thereby improving the density of the matrix (Figure 11).

**Figure 12:**
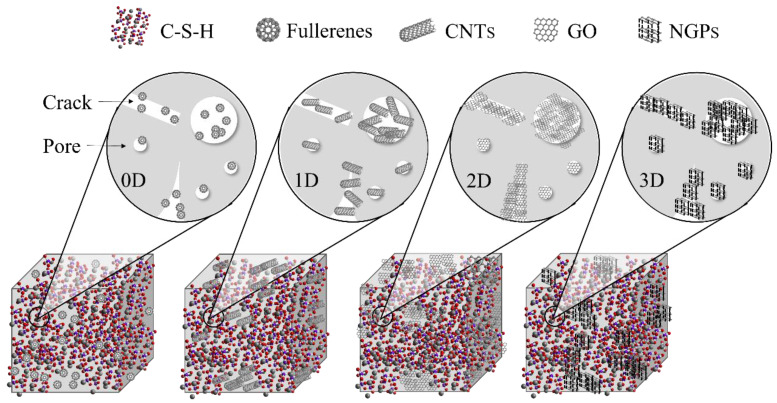
Mechanism of different dimensions of carbon-based nanomaterials on cementitious composites.

Figure 12 shows the mechanism of zero—three dimensions of carbon-based nanomaterials on cementitious composites. Moreover, 0D fullerene is distributed in the form of points inside the cementitious composites and is relatively dispersed. Additionally, 1D CNTs grow in the form of lines inside cementitious composites or are composited with matrix materials. This will hinder the development of cracks during the stressing process of cementitious composites and take the lead in improving the tensile properties of the material. Also, 2D GO grows in the form of surfaces inside the cementitious composites, and the contact area with the matrix is greatly increased. It can comprehensively improve the mechanical properties of cementitious composites. However, 3D NGPs form a three-dimensional network of internal components in cementitious composites. As far as current research results are concerned, the improvement effect of 3D NGPs mechanical properties is almost the same as that of 2D GO. The possible reason is that 3D carbon-based nanomaterials have higher requirements for dispersion technology.

## 7. Conclusions

This article provides a detailed analysis of the current literature on the performance of cementitious composites reinforced with carbon-based nanomaterials. The main conclusions drawn from this research are as follows:

(1) The five-parameter system used to evaluate the environmental effects of carbon-based nanomaterials shows that 2D GO has the best performance, followed by 3D NGPs, 1D CNTs, and 0D fullerenes. 

(2) GO stands out for its lower energy consumption (120–140 MJ/kg) and CO_2_ emissions (0.17 kg/kg). 

(3) In terms of improving the mechanical properties of cementitious composites, GO exhibits excellent performance, followed by 3D NGPs, 1D CNTs, and 0D fullerenes.

(4) The optimal dosage of GO to improve the compressive strength of cementitious composites is between 0.01 and 0.05 wt%, and the maximum enhancement rate is approximately 50%. The reason why the mechanical properties of cementitious composites are improved is that the high specific surface area of GO promotes cement hydration and fills pores. More importantly, the high specific surface area of GO is beneficial to the strong adhesion between GO and the matrix and prevents cracks from expanding under load.

A comprehensive literature review has been conducted in this study. However, it is important to acknowledge its limitations. Specifically, the dispersion technology of 3D NGP_S_ is not yet mature, resulting in the mechanical properties of cementitious composites needing to be further improved. Additionally, the high cost of 2D GO hinders its widespread application. In future work, it is necessary to further develop advanced dispersion equipment or find low-cost carbon sources to systematically address these limiting factors and delve into the untapped potential of carbon-based nanomaterials in cementitious composites, especially related to their performance improvement and environmental impact.
